# Combined Downregulation of Insulin and Mitochondrial Signaling Leads to Synergistic Life- and Healthspan Extension in Caenorhabditis Elegans

**DOI:** 10.1093/geroni/igaf122.968

**Published:** 2025-12-31

**Authors:** Sinclair Emans, Nicole Sthur, Sophia DiPaola, Zoe Nathwani, Alexander Soukas

**Affiliations:** Harvard Medical School, Boston, Massachusetts, United States; University of Southern California, Los Angeles, California, United States; Eckerd College, St. Petersburg, Florida, United States; Harvard University, Boston, Massachusetts, United States; Massachusetts General Hospital, Boston, Massachusetts, United States

## Abstract

The Caenorhabditis elegans insulin/IGF-1 signaling (IIS) pathway functions as both a rheostat for nutrient levels and as an evolutionarily conserved mechanism likely to shed light on its functions and regulation in higher organisms. Early studies in C. elegans demonstrated that IIS receptor homolog daf-2 downregulation led to a drastic increase in lifespan, from a maximum of 25 to 60 days. Over the following decades many other manipulations were shown to increase lifespan in worms including interventions in diet, AMPK, mTOR signaling, and mitochondrial homeostasis, to name just a few. However, combinatorial lifespan studies still remain few and far between. Herein, we demonstrate that combining a daf-2 hypomorph with adenine nucleotide translocase 1.1 (ant-1.1) knockdown leads to a four-fold increase in lifespan and a concomitant healthspan increase. We further demonstrate that this increase is not dependent on the mitochondrial unfolded protein response. Moreover, we show that a distinctive transcriptional signature could explain this distinctive synergistic phenomenon. In sum, we describe a combinatorial treatment that can quadruple lifespan and produce a similar healthspan increase through a potentially unique transcriptional program.

